# The multifaceted role of Nrf2 in mitochondrial function

**DOI:** 10.1016/j.cotox.2016.10.002

**Published:** 2016-12

**Authors:** Kira M. Holmström, Rumen V. Kostov, Albena T. Dinkova-Kostova

**Affiliations:** 1 BioMediTech and Tampere University Hospital, University of Tampere, Tampere, Finland; 2 Institute of Biotechnology, University of Helsinki, Helsinki, Finland; 3 Division of Cancer Research, School of Medicine, University of Dundee, Dundee, Scotland, UK; 4 Department of Pharmacology and Molecular Sciences, Johns Hopkins University School of Medicine, Baltimore, MD, USA; 5 Department of Medicine, Johns Hopkins University School of Medicine, Baltimore, MD, USA

**Keywords:** Glucoraphanin, Keap1, Mitohormesis, Mitophagy, Neurodegenerative disease, Nrf, PMI, RTA-408, Stem cells, Sulforaphane

## Abstract

The transcription factor nuclear factor erythroid 2 p45-related factor 2 (Nrf2) is the master regulator of the cellular redox homeostasis. Nrf2 target genes comprise of a large network of antioxidant enzymes, proteins involved in xenobiotic detoxification, repair and removal of damaged proteins, inhibition of inflammation, as well as other transcription factors. In recent years it has emerged that as part of its role as a regulator of cytoprotective gene expression, Nrf2 impacts mitochondrial function. Increased Nrf2 activity defends against mitochondrial toxins. Reduced glutathione, the principal small molecule antioxidant in the mammalian cell and a product of several of the downstream target genes of Nrf2, counterbalances mitochondrial ROS production. The function of Nrf2 is suppressed in mitochondria-related disorders, such as Parkinson's disease and Friedrich's ataxia. Studies using isolated mitochondria and cultured cells have demonstrated that Nrf2 deficiency leads to impaired mitochondrial fatty acid oxidation, respiration and ATP production. Small molecule activators of Nrf2 support mitochondrial integrity by promoting mitophagy and conferring resistance to oxidative stress-mediated permeability transition. Excitingly, recent studies have shown that Nrf2 also affects mitochondrial function in stem cells with implications for stem cell self-renewal, cardiomyocyte regeneration, and neural stem/progenitor cell survival.

## Highlights

• Nrf2 activity defends against mitochondrial toxins.
• The function of Nrf2 is suppressed in mitochondria-related disorders.
• Nrf2 deficiency leads to impaired mitochondrial fatty acid oxidation, respiration and ATP production.
• Small molecule activators of Nrf2 support mitochondrial integrity.
• Nrf2 affects mitochondrial function in stem cells.

## Graphical abstract

**Figure:**
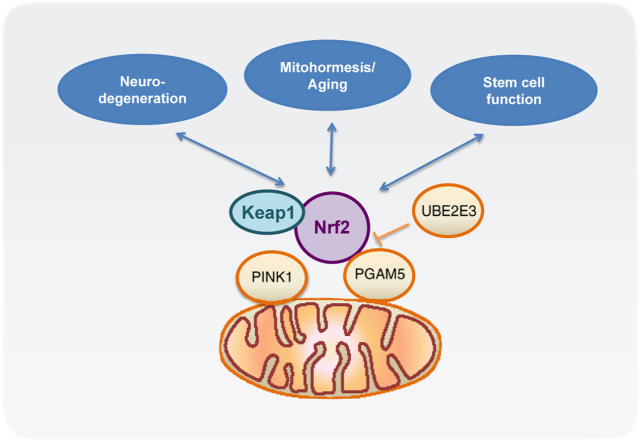

## 1 Introduction

The mitochondria are known as the powerhouse of the cell. The process of providing the cell with the bulk of its energy is intimately linked to the production of reactive oxygen species (ROS) during oxidative phosphorylation. In most cells, the mitochondria and NADPH oxidase are the main sources of ROS. Our understanding of the role of ROS within the cell is becoming increasingly complex. The traditional view of ROS simply being a harmful by-product of respiration is giving way to a more intricate picture where the role of ROS as an important signaling molecule is emerging 1, 2. It is however becoming evident that an imbalance in the generation of ROS is a common feature in several disease states, ranging from neurodegeneration and diabetes to cardiovascular disease and cancer [3].

As the master regulator of the cellular redox homeostasis, the cap ‘n’ collar basic leucine zipper (CNC-bZip) transcription factor, nuclear factor erythroid 2 p45-related factor 2 (Nrf2) is well equipped to counterbalance the mitochondrial ROS production and is critical for maintaining the redox balance in the cell [4]. Following exposure to oxidants or electrophiles, Nrf2 accumulates in the nucleus. There, it binds to antioxidant response elements (ARE) in the upstream regulatory regions of genes encoding detoxification and antioxidant enzymes, leading to their enhanced transcription 4, 5, 6. Work from our laboratories and the laboratories of other investigators, has shown that the status of Nrf2 activity affects mitochondrial function, and this has been reviewed 7, ∗8, 9, 10. The current *opinion article* briefly summarizes the available experimental evidence and provides an update of the most recent findings in this area.

## 2 Nrf2 regulation

**Figure 1:**
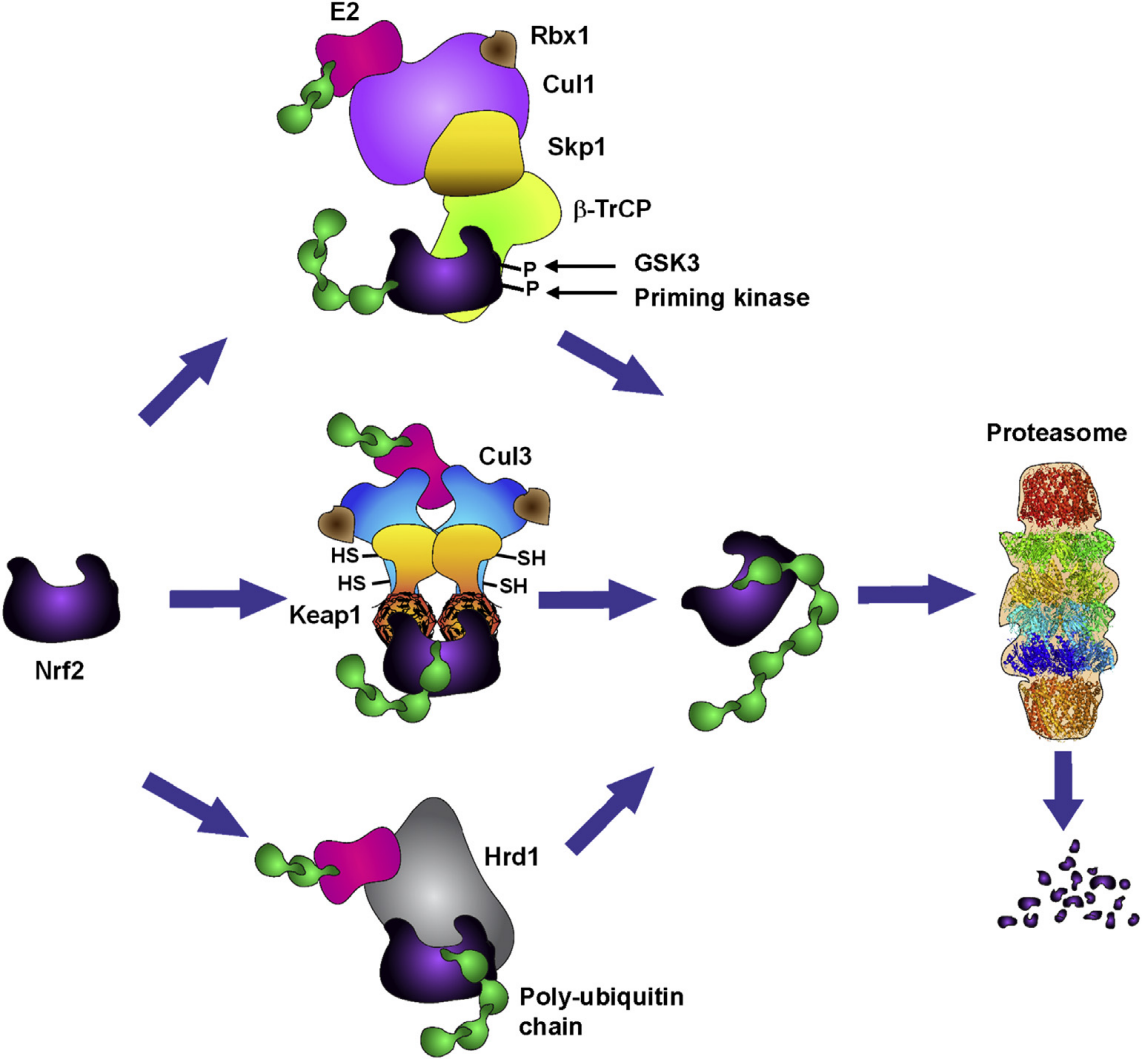
**Regulation of Nrf2 under homeostatic conditions**. Nrf2 is a short-lived protein that is continuously targeted for ubiquitination and proteasomal degradation. Three known ubiquitin ligase systems mediate the degradation of Nrf2: Kelch-like ECH associated protein 1 (Keap1), a substrate adaptor protein for Cullin3 (Cul3)/Rbx1-based Cullin–RING E3 ubiquitin ligase and a cysteine-based sensor for Nrf2 inducers; β-transducin repeat-containing protein (β-TrCP), a substrate adaptor for Skp1–Cullin1 (Cul1)/Rbx1-based Cullin–RING E3 ubiquitin ligase; and the E3 ubiquitin ligase Hrd1 which resides in the endoplasmic reticulum (ER). The relative contributions of these systems towards the degradation of Nrf2 depend on the specific conditions. Degradation mediated by Keap1 requires reduced state of its cysteine sensors. Degradation mediated by β-TrCP requires prior phosphorylation of Nrf2 by glycogen synthase kinase 3 (GSK3). Degradation mediated by Hrd1 occurs during ER stress.

Under basal conditions, Nrf2 is rapidly turned over, and the function of Nrf2 is primarily regulated by controlling the protein levels of the transcription factor through ubiquitination and proteasomal degradation. There are three known ubiquitin ligase systems that are responsible for Nrf2 degradation (Figure 1). The first discovered and most studied is the Kelch-like ECH-associated protein 1 (Keap1)–Cullin3 (Cul3)/Rbx1 11, 12, 13. As a negative regulator of Nrf2 [14], Keap1 serves as a substrate adaptor protein for the ubiquitin ligase Cul3/Rbx1. Keap1 binds Nrf2 in the cytoplasm and targets the transcription factor for ubiquitination and proteasomal degradation, maintaining Nrf2 at a low steady state level. Oxidants and electrophiles react with cysteine sensors within Keap1 15, 16, 17, causing a conformational change 18, ∗19 and the inability of Keap1 to target Nrf2 for degradation [20]. This allows free Nrf2 to accumulate and translocate to the nucleus where it binds to a small Maf protein, activating the expression of its target genes 21, ∗∗22. Nrf2 is also subject to degradation following phosphorylation by glycogen synthase kinase 3 (GSK3) via β-transducin repeats-containing protein (β-TrCP)-Cul1-based ubiquitin ligase ∗23, 24. The most recently described ubiquitin-dependent system involved in Nrf2 degradation is the E3 ubiquitin ligase synoviolin (Hrd1), which resides in the endoplasmic reticulum [25].

Besides regulation of Nrf2 through its degradation, the function of the transcription factor is also controlled through the spatial distribution of both Nrf2 and Keap1. There are three pools of Nrf2 within the cell. In addition to the predominant cytoplasmic pool, there is a nuclear pool of Nrf2, the redistribution of which is controlled in part by Keap1-mediated degradation and by Nrf2 nuclear import signals and mediators [26]. Nrf2 and Keap1 have also been detected at the outer mitochondrial membrane, tethered to the mitochondrial phosphatase phosphoglycerate mutase family member PGAM5 [27]. The three pools of Nrf2 are highly dynamic and subjected to a further fine-tuned regulation. Thus, it has been reported that the ubiquitin-conjugating enzyme UBE2E3 and its nuclear import receptor importin 11 regulate Nrf2 distribution and activity, by restricting the transcription factor from partitioning to the mitochondria and limiting its repression by nuclear Keap1 [28].

## 3 Nrf2 and the cellular redox homeostasis

**Figure 2:**
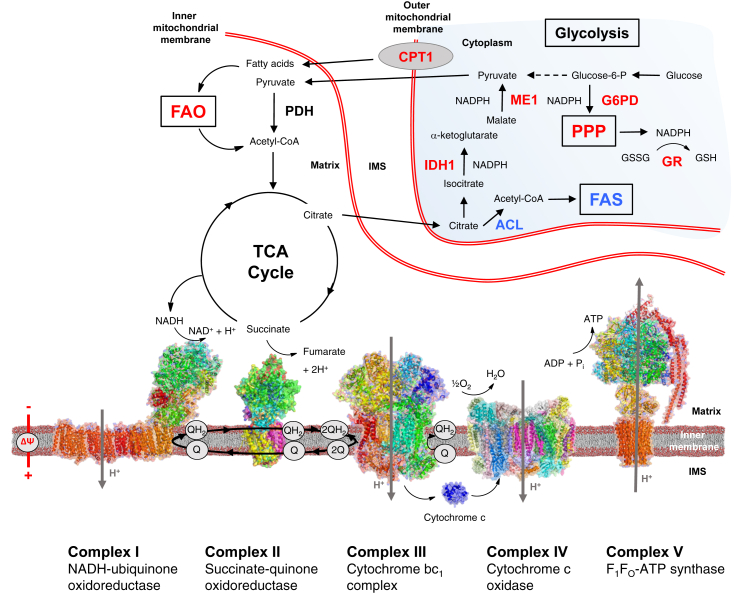
**Nrf2 affects mitochondrial function at multiple levels**. Nrf2 activation increases the mitochondrial membrane potential (ΔΨ), the availability of substrates for respiration, and ATP production. Nrf2 positively regulates the levels of NADPH by enhancing the expression of genes encoding glucose-6-phosphate dehydrogenase (G6PD), the enzymes of the pentose phosphate pathway (PPP), malic enzyme 1 (ME1) and isocitrate dehydrogenase 1 (IDH1). In addition to NADPH, ME1 regenerates pyruvate, which can cycle back to the mitochondria. Nrf2 also regulates the levels of GSH by enhancing the expression of genes encoding enzymes involved in its biosynthesis and regeneration from its oxidized form, GSSG, including glutathione reductase (GR). Nrf2 negatively regulates ATP-citrate lyase (ACL), acetyl-CoA carboxylase, fatty acid synthase, and stearoyl CoA desaturase, four critical enzymes involved in fatty acid synthesis (FAS). A decrease in the levels of malonyl-CoA may increase mitochondrial fatty acid oxidation (FAO) by relieving its inhibitory function on carnitine palmitoyltransferase 1 (CPT1), which mediates the transport of long-chain fatty acids into the mitochondria. The red and the blue colors indicate positive and negative regulation by Nrf2, respectively. The presentation of the structure of each respiratory complex is adapted from reference [103]. IMS, mitochondrial intermembrane space.

Since its discovery in the mid-1990s ∗∗22, ∗29, Nrf2 has been extensively studied. The number of publications on Nrf2 has exceeded 7000, and continues to increase exponentially (http://www.ncbi.nlm.nih.gov/pubmed/?term=nrf2). Nrf2 has been associated with cytoprotective functions in animal models of a range of human disease conditions, and has been implicated in the regulation of over 600 target genes [30]. Nrf2 targets include antioxidant enzymes, proteins involved in xenobiotic metabolism and clearance, protection against heavy metal toxicity, inhibition of inflammation, repair and removal of damaged proteins, as well as other transcription and growth factors [31]. Nrf2 regulates the expression of γ-glutamyl cysteine ligase catalytic (GCLC) and modulatory (GCLM) subunits, glutathione reductase (GR) 21, 30, 32, 33, 34, 35, as well as the four enzymes [i.e. malic enzyme 1 (ME1), isocitrate dehydrogenase 1 (IDH1), glucose-6-phosphate dehydrogenase (G6PD), and 6-phosphogluconate dehydrogenase (6PGD)] that are responsible for the generation of NADPH 36, 37, ∗∗38, 39, ∗40, all of which are involved in the biosynthesis and maintenance of reduced glutathione (GSH). In turn, GSH, the principal small molecule antioxidant in the mammalian cell, counterbalances the production of ROS. In more recent years, it has emerged that one of the important functions of Nrf2 is to modulate mitochondrial function, as part of its role as a master regulator of cytoprotective gene expression and the cellular redox homeostasis (Figure 2). The evidence for this is two fold. First, it has been shown that the Nrf2 pathway is upregulated and is involved in protection against mitochondrial toxins. Early work noted that increased Nrf2 activity enhanced resistance to mitochondrial toxins such as the complex I inhibitor rotenone or the complex II inhibitor 3-nitropropionic acid *in vitro* and *in vivo* 41, 42, ∗43. Second, Nrf2 function has been reported to be impaired in mitochondria-related disorders, whereas Nrf2 activation has beneficial effects. For example, the Nrf2 pathway is suppressed in Parkinson's disease patient derived olfactory neurosphere cells [44], and Nrf2 activation restores the glutathione levels in these cells [45]. This Nrf2 suppression is especially prominent in Friedrich's ataxia where Nrf2 activation upon oxidative stress was found to be blocked in patient fibroblasts [46].

## 4 Nrf2 and mitochondrial homeostasis

In 2008, Lo and colleagues reported that Keap1 associates with PGAM5, establishing a physical link to mitochondria [27]. That same year, an association between Nrf2 and mitochondrial biogenesis was found in cardiomyocytes, where Nrf2 stimulates the biogenesis program through activation of nuclear respiratory factor-1 (NRF-1) [47]. This has since been confirmed in *in vivo* studies [48]. What we have been interested in establishing is a more direct involvement of Nrf2 in modulating mitochondrial function (recently reviewed in 7, ∗8, 10). We showed that respiration and ATP levels are decreased in cells and mitochondria isolated from Nrf2-knockout (Nrf2-KO) mice, while they are increased in their Keap1-knockout (Keap1-KO) and Keap1-knockdown (Keap1-KD) counterparts ∗∗49, 50. Similarly, mitochondrial fatty acid oxidation is impaired in cells and mitochondria isolated from Nrf2-KO mice [51]. This could potentially be the reason for the higher accumulation of triglycerides in the liver upon fasting in these mice [52]. As the activities of the respiratory enzymes are not impaired [49], the decrease in respiration and ATP levels under conditions of Nrf2 deficiency argue that the main limitation is substrate availability.

**Figure 3:**
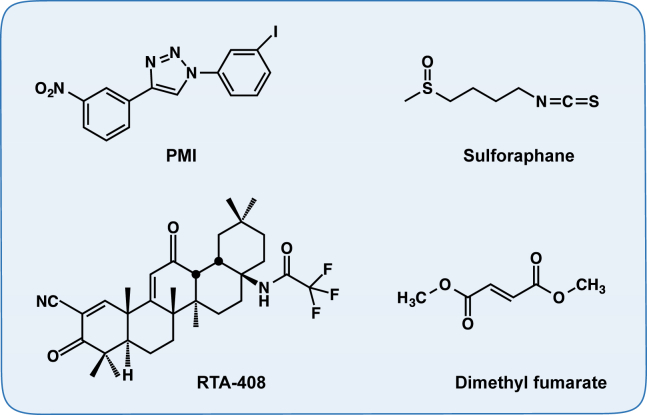
**Examples of small-molecule Nrf2 activators that improve mitochondrial function and integrity**. Chemical structures of 1-(3-iodophenyl)-4-(3-nitrophenyl)-1,2,3-triazole (PMI), 1-isothiocyanato-(4*R*)-(methylsulfinyl)butane (sulforaphane), N-(11-cyano-2,2,6a,6b,9,9,12a-heptmethyl-10,14-dioxo-1,3,4,5,6a,6b,7,8,8a,9,10,12a,14,14a,14b-hexadecahydro-2H-picen-4a-yl)-2-2-difluoro-propionamide (RTA-408, Omaveloxolone) and dimethyl fumarate (BG-12, Tecfidera).

Mitochondrial integrity is key to overall mitochondrial functionality. Mitophagy has emerged as a way to maintain the organelle integrity, by selectively removing damaged mitochondria [53]. One of the critical players involved in this process is the autophagic adaptor protein sequestosome-1 (SQSTM1/p62) [54]. p62 interacts with the Nrf2-binding site on Keap1, competing with Nrf2 for binding ∗55, ∗56. This interaction is further enhanced by phosphorylation ∗57, 58. Therefore, increased free p62 levels activate the Nrf2 pathway. p62 is also an Nrf2-target gene, thus creating a positive regulatory loop ∗55, ∗56. An Nrf2-dependent small-molecule mitophagy inducer (p62-mediated mitophagy inducer – PMI) (Figure 3) was recently discovered. PMI directly disrupts the Nrf2-Keap1 interaction [59] and induces mitophagy independently of dissipation of the mitochondrial membrane potential and the mitochondrial serine/threonine-protein kinase PTEN-induced kinase 1 (PINK1)/Parkin pathway [60].

When mitochondrial integrity is lost beyond repair, the mitochondria can undergo permeability transition to induce cell death [61]. Induction of Nrf2 using the isothiocyanate sulforaphane (Figure 3) ∗∗62, 63 confers resistance to redox-regulated permeability transition [64], suggesting a further role for the Nrf2 pathway in maintaining mitochondrial integrity.

## 5 Nrf2, mitochondrial function and neurological conditions

Neurodegenerative disorders are commonly characterized by oxidative stress, mitochondrial dysfunction and protein misfolding, making them ideal targets for Nrf2 activator mediated therapy (reviewed in 10, 65, 66). Nrf2 activation has long been shown to be cytoprotective in both toxicological as well as genetic models of neurodegeneration *in vitro* and *in vivo* ∗67, 68, ∗69, ∗70, 71, ∗72, 73, 74, 75, ∗∗76. More recently, we have reported that treatment with the Nrf2 inducers RTA-408, a synthetic cyanoenone triterpenoid, or with the naturally occurring isothiocyanate sulforaphane (Figure 3) restored the mitochondrial membrane potential and protected against dopamine toxicity in primary co-cultures of midbrain neurons and astrocytes isolated from PINK1-KO mice, a model of hereditary early-onset Parkinson's disease [8]. A wide variety of small molecule activators of the Nrf2 pathway have been established and tested in both *in vitro* and *in vivo* models of neurodegenerative diseases, including multiple sclerosis, Parkinson's, Huntington's and Alzheimer's disease (recently reviewed in [77]), showing great promise as potential therapeutic agents. Sulforaphane has shown protective effects in a number of rodent models of neurological conditions (Table 1). Several Nrf2 activators are undergoing clinical trials; one of them, BG-12 (Tecfidera), has already entered clinical practice. BG-12 is an oral formulation of the Nrf2 inducer dimethyl fumarate (Figure 3), which is being used for the treatment of relapsing – remitting multiple sclerosis in humans 78, 79. Currently, the Nrf2 activator RTA-408 (Figure 3) is being tested for treatment of Friedrich's ataxia (ClinicalTrials.gov, NCT02255435). The potential of GSK3 inhibitors (Tideglusib) in Alzheimer's disease was explored in a small Phase II clinical trial. Although in this trial no overall statistically significant clinical benefit for the drug was found, it was noted that there was a significant decrease in the levels of β-secretase 1 (BACE1) in cerebrospinal fluid in a subgroup of patients [80].

A recent study reported the ability of sulforaphane to improve social interaction and verbal communication, reversing abnormal behavior in young men with autism spectrum disorder [81]. Interestingly, granulocytes of children with autism exhibit defects in oxidative phosphorylation and reduced gene expression of Nrf2 [82]. In healthy human subjects, metabolic profiling after a dietary intervention with broccoli as a source of glucoraphanin, the precursor of sulforaphane, has indicated enhanced integration of fatty acid oxidation with the activity of the TCA cycle [83]. Taken together, these studies suggest that sulforaphane-mediated Nrf2 activation may lead to improved mitochondrial function and thus contribute to reversal of the behavioral abnormalities in autism.

## 6 Nrf2 and mitohormesis

An interesting concept that has grown in popularity is the involvement of Nrf2 signaling in hormesis. Hormesis refers to the exposure to low levels of stress such as ROS, which will prime the cell or organism to better handle future insults [84]. Mitohormesis more specifically suggests that the mitochondria might be essential for this process [85]. Nrf2 has been suggested multiple times to have hormetic potential 84, 86, 87. This has been extensively discussed in the context of nutritional antioxidants and dietary restriction [88], where it has been shown that Nrf2 is in part responsible for the beneficial effects of dietary restriction through activation of the phase 2 response. SKN-1, the Nrf ortholog in the nematode *Caenorhabditis elegans*, has been shown to be a longevity factor 89, 90. SKN-1 activation reduces the accumulation of ROS and increases proteasome activity, stress resistance, and lifespan 89, 91. The exact mechanism is not fully understood, but SKN-1 is responsible for mitochondria-associated redox signaling [90], and for coupling proline catabolism with fatty acid oxidation during limited nutrient availability [92].

Most recently, Nrf2 activation was linked to lithium-mediated lifespan extension in *Drosophila melanogaster* [93]. Lithium inhibits GSK3, and this inhibition stabilizes and activates Nrf2 (Figure 1), thus extending the lifespan of the flies, specifically at low doses. As with any hormetic response, excessive levels of the toxin, and even excessive Nrf2 activation, has detrimental consequences and decreases lifespan. This is in line with the phenotype of the Keap1-KO mice, which die postnatally from hyperkeratosis of the esophagus due to constitutive Nrf2 activation [94], and with the reduced longevity due to prolonged Nrf2 overexpression in transgenic *Drosophila melanogaster* [95].

## 7 Emerging role of Nrf2 in mitochondrial function in stem cells

Although not an entirely novel concept, 2016 has seen a surge in high impact publications that have explored the relationship between Nrf2 and mitochondrial function in the context of stem cell biology. Decreased levels of Nrf2 were shown to correlate with the decrease in regenerative capacity of subventricular zone neural stem/progenitor cells (NSPCs) in the rat [96]. Intriguing work by Khacho and colleagues [97] suggests that dynamic changes in the mitochondrial network during neural stem cell development induce ROS-dependent Nrf2-mediated transcriptional activation of cell differentiation. The metabolic reprogramming from oxidative phosphorylation to glycolytic energy production that takes place during the induction of pluripotent stem cells differentiation is also dependent on ROS-mediated Nrf2 activation ∗∗98, 99. In the heart, Nrf2 is necessary for neonatal myocardial regeneration after apex resection by activating paired-like homeodomain transcription factor 2 (Pitx2), which then activates antioxidant genes as well as components of the electron transport chain [100].

The age-related decline in the regenerative function of neural stem/progenitor cells has been causally linked to decreased expression of Nrf2 [98]. A recent report found that Nrf2 activity is impaired in the premature aging disorder Hutchinson-Gilford progeria syndrome (HGPS) due to progerin sequestration of Nrf2, leading to subnuclear mislocalization of the transcription factor [101]. Reactivation of the Nrf2 pathway reverses the cellular phenotype, including key phenotypes of the disease, such as reduced viability of mesenchymal stem cells [101] and impaired autophagy [102], while inactivation of the pathway recapitulates some of the aging phenotypes in HGPS. Together, these studies show that Nrf2 is an important player in stem cell biology and cell senescence, and implicate its role in mitochondrial function as a possible mechanistic link.

## 8 Concluding remarks and future directions

Work from a number of independent laboratories has convincingly demonstrated that the status of Nrf2 activity affects many aspects of mitochondrial physiology, including mitochondrial biogenesis, fatty acid oxidation, respiration, ATP production, redox homeostasis, as well as the structural integrity and dynamics of this essential organelle. In parallel to recognizing that many human pathological conditions and aging are associated with mitochondrial dysfunction, it is becoming increasingly apparent that this often coincides with suppressed Nrf2 signaling. Most excitingly, the ability to reactivate Nrf2 by pharmacological agents is a promising strategy for the prevention or treatment of chronic degenerative diseases and for achieving healthy aging. Importantly, pharmacological Nrf2 activators include phytochemicals (e.g. sulforaphane) that are present in plants, such as cruciferous vegetables, which have been an important part of the human diet for centuries, and are largely responsible for the health-promoting effects of plant-rich diets. As both insufficient as well as persistently high Nrf2 activity can have detrimental consequences, it will be critical to understand what is the appropriate “dose” of Nrf2 activity that would restore the balance and correct the pathological phenotypes.

## Figures and Tables

**Table 1:** Protective Activity of Sulforaphane and Its Glucoraphanin Precursor in Rodent Models of Neurological Conditions.

Condition/disease	Species/strain	Damaging agent	Sulforaphane dose	Efficacy endpoints	References
Brain injury	Rat ♂ Sprague Dawley	Controlled cortical impact	5 mg/kg, i.p., 6 h post-injury	Increase in AQP4 in penumbra; increase in GPx in cortex; increase in GSTα3 and HO-1 in brain microvessels; decrease in loss of tight junction proteins and endothelial cells; decrease in blood-brain barrier permeability and cerebral edema	Zhao et al. (2005, 2007) 104, 105
Brain injury	Rat ♂ Sprague Dawley	Controlled cortical impact	5 mg/kg, i.p., 15 min post-injury	Increase in Nrf2, NQO1 and HO-1 in cortex; decreased oxidative damage to lipids, proteins and DNA; decreased brain contusion volume and cortical neuronal death; improved neurologic function	Hong et al. (2010) [106]
Brain injury	Mouse ♂ C57BL/6 WT and Nrf2^−/−^	Controlled cortical impact	5 mg/kg, i.p., 6 h post-injury	Decrease in blood-brain barrier permeability in WT mice; Nrf2^−/−^ mice more sensitive than WT mice & no protection by SFN	Zhao et al. (2007) [105]
Brain injury	Mouse ♂ ICR WT and Nrf2^−/−^	Controlled cortical impact	5 mg/kg, i.p., 15 min post-injury	Nrf2^−/−^ mice more sensitive than WT mice & no protection by SFN	Hong et al. (2010) [106]
Brain injury	Rat ♂ Wistar	Subarachnoid hemorrhage	5 mg/kg, i.p., 30 min, 12 h, and 36 h after blood injection	Increase in Nrf2, NQO1, GSTα1 and HO-1 in cortex; decrease in cerebral edema, blood-brain barrier impairment, cortical apoptosis, and motor deficits	Chen et al. (2011) [107]
Spinal cord injury	Mouse ♂ ICR WT and Nrf2^−/−^	Contusion injury (vascular clip, 10 g)	5 mg/kg, i.p., 1 h after injury	Decrease in MMP9 and TNFα, vascular permeability changes, inflammatory damage, histologic injury, dying neurons count, and spinal cord edema; enhanced hindlimb locomotor function; Nrf2^−/−^ mice more sensitive than WT mice & no protection by SFN	Mao et al. (2010, 2011) 108, 109
Spinal cord injury	Rat ♀ Fischer	Contusion injury (weight drop, 10 g)	5 mg/kg, i.p., 15 min after injury, then once a day for 3 days	Increase in Nrf2 and GCLC in spinal cord 1 day after injury; decrease in IL-1β, TNFα, IκBα phosphorylation, and contusion volume; improvement in coordination	Wang et al. (2012) [110]
Spinal cord injury	Rat ♀ Sprague-Dawley	Contusion injury (200 kdyn)	10 or 50 mg/kg, i.p., 10 min and 72 h after injury	Increase in NQO1 and HO-1, and decrease in MMP9 in spinal cord 4 h after injury; decrease in urinary MIF activity; increase in serotonergic axons caudal to the lesion; enhanced hindlimb locomotor function	Benedict et al. (2012) [111]
Stroke	Rat ♂ Long–Evans	Temporary common carotid/middle cerebral artery occlusion	5 mg/kg, i.p., 15 min post-ischemia	Increase in HO-1 in brain; decrease in infarct volume	Zhao et al. (2006) [112]
Alzheimer's disease	Mouse ♂ ICR	Aβ(1-40) injection, i.c.v.	30 mg/kg/day, i.p., from day −1 to day 4 post-Aβ	Decrease in impairment of working and contextual memory; no effect on amyloidogenesis	Kim et al. (2013) [74]
Parkinson's disease	Mouse ♂ C57BL/6 WT and Nrf2^−/−^	MPTP (for 5 consecutive days starting on day 0)	50 mg/kg, i.p., on day −1 (2 doses, 8 h apart); then daily doses on day 1, 3 and 5	Increase in NQO1, HO-1, GCLC and GCLM in striatum and ventral midbrain; decrease in loss of dopaminergic neurons, astrogliosis and microgliosis; decrease in pro-inflammatory mediators (IL6 and TNFα); Nrf2^−/−^ mice more sensitive than WT mice & no protection by SFN	Rojo et al. (2010), Innamoratoet al. (2010), and Jazwa et al. (2011) ∗70, ∗72, 113
Parkinson's disease	Mouse ♂ C57BL6/SJL	MPTP		Nrf2^−/−^ mice more sensitive than WT mice; protection by Nrf2 overexpression or Keap1 (by siRNA) downregulation	Chen et al. (2009) and Williamson et al. (2012) 68, ∗69
Parkinson's disease	Mouse ♂ C57BL/6	6-Hydroxy-dopamine-induced lesion	5 mg/kg, i.p., twice a week for 4 weeks starting after lesion induction	Decrease in motor function deficits; decrease in degeneration of dopaminergic neurons and DNA fragmentation; increase in GSH and GR	Morroni et al. (2013) [114]
Parkinson's disease	Mouse ♂ C57BL/6	Rotenone	50 mg/kg, i.p., every other day before rotenone for 60 days	Increase in NQO1, HO-1 and LC3-II in cortex and striatum compared to rotenone treatment; decrease in rotenone-induced oxidative damage; decrease in loss of dopaminergic neurons; decrease in motor function deficits	Zhou et al. (2016) [115]
Huntington's disease	Rat ♂ Wistar	2,3-Pyridine-dicarboxylic acid (quinolinic acid)	5 mg/kg, i.p., 24 h and 5 min before intrastriatal infusion of quinolinic acid	Increase in GSH, GR, and GPx; decrease in oxidized proteins, mitochondrial dysfunction, striatal degeneration and circling behavior	Santana-Martínez et al. (2014) and Luis-García et al. (2016) 116, 117
Depression	Mouse ♂ Swiss and C57BL/6 WT and Nrf2^−/−^	LPS	1 mg/kg, i.p., for 7 consecutive days before and the day after LPS	Compared to WT mice, decrease in dopamine and serotonin levels in prefrontal cortex, retraction of astroglial processes, increased microgliosis and depressive phenotype of Nrf2^−/−^ mice without LPS; Increase in HO-1, GCLM and BDNF and decrease in iNOS by SFN in hippocampus of in WT mice with LPS; Improved depressive-like behavior in WT mice with LPS	Martín-de-Saavedra et al. (2013) [118]
Depression	Mouse ♂ ICR	Acute stress Chronic stress (28 days)	1, 3, or 10 mg/kg/day, i.p., for 14 days 10 mg/kg/day, i.p., for 14 days starting on day 14	Reversal of depressive- and anxiety-like behavior Decrease in pro-inflammatory mediators (IL6 and TNFα) and serum corticosterone and adrenocorticotropic hormone levels; reversal of depressive- and anxiety-like behavior	Wu et al. (2016) [119]
Depression	Mouse ♂ C57BL/6 WT and Nrf2^−/−^	Repeated social defeat stress for 10 days	10 mg/kg, i.p., 30 min before defeat stress or 0.1% dietary glucoraphanin	Attenuation of decreased levels in Keap1, Nrf2, BDNF, p-TrkB, and depression-like behavior; Nrf2^−/−^ mice more sensitive than WT mice	Yao et al. (2016) [120]
Multiple sclerosis	Mouse ♀ C57BL/6	(MOG)_35–55_ immunization, followed by Pertussis toxin	50 mg/kg, i.p., every other day for 22 days	Inhibition of development and severity of experimental autoimmune encephalomyelitis; increase in HO-1 and NQO1, and decrease in oxidative stress in brain; decrease in MMP9, inflammatory infiltration and demyelination in spinal cord; improved distribution of claudin-5 and occluding; preservation of the blood–brain barrier; inhibition of antigen-specific Th17 responses and enhanced IL10 responses	Li et al. (2013) [121]
Multiple sclerosis	Mouse ♂ C57BL/6	MOG35-55 immunization, followed by Pertussis toxin	10 mg/kg/day, i.p., myrosinase-activated glucoraphanin beginning 1 week before immunization	Decrease in inflammation (NFkB translocation and IL1β) and apoptosis (Bax and caspase 3) in spinal cord; protection against body weight loss	Giacoppo et al. (2013) [122]

**Abbreviations:** AQP4, aquaporin 4; BDNF, brain-derived neurotrophic factor; GCLC, glutamate cysteine ligase catalytic subunit; GCLM, glutamate cysteine ligase modulatory subunit; GPx, glutathione peroxidase; GSH, reduced glutathione; GST, glutathione *S*-transferase; HO-1, heme oxygenase 1; IκBα, nuclear factor kappa-light-chain-enhancer of activated B cells inhibitor, α; IL, interleukin; LC3, microtubule-associated protein light chain 3; LPS, lipopolysaccharide; MIF, macrophage inhibitory factor; MMP9, matrix metalloproteinase 9; MOG, myelin oligodendroglial glycoprotein peptide; MPTP, methyl-4-phenyl-1,2,3,6-tetrahydro-pyridine; NFkB, nuclear factor kappa-light-chain-enhancer of activated B cells; NQO1, NAD(P)H:quinone oxidoreductase 1; SFN, sulforaphane; TNFα, tumor necrosis factor α; p-TrkB, phosphorylated tropomyosin-receptor-kinase B.
